# Evaluating the impact of COVID-19 on ex-vessel prices using time-series analysis

**DOI:** 10.1007/s12562-021-01574-x

**Published:** 2022-01-24

**Authors:** Keita Abe, Gakushi Ishimura, Shinya Baba, Shota Yasui, Kosuke Nakamura

**Affiliations:** 1 Centre for Applied Research at NHH, Helleveien 30, 5045 Bergen, Norway; 2 Iwate University and National Institute of Environmental Studies, Morioka, Japan; 3 Logics of Blue, Hyogo, Japan; 4 CyberAgent, Inc, Tokyo, Japan; 5 Iwate University, Morioka, Japan

**Keywords:** Ex-vessel price formation, Time-series analysis, COVID-19’s impact on fisheries, Forecasting

## Abstract

The spread of coronavirus disease 2019 (COVID-19) and subsequent lockdown measures have impacted economies and industries worldwide. The fisheries industry witnessed a sharp decline in demand and a slump in fish prices due to its dependence on the food service industry. It is important to quantitatively assess those fish species affected most and the extent of the pandemic’s impact on them, to take specific countermeasures. We propose a time-series analysis as an alternative to the current practice of using ad hoc year-on-year comparisons. Although the pandemic makes it difficult to construct a counterfactual approach due to the lack of an appropriate control group, we use time-series forecasting to simulate normal conditions using pre-pandemic data. In Tokyo, the unit price of fish species that were negatively impacted by the food services industry dropped by 12.65% to 14.64%, and by 26.08% to 28.22% after the declaration of a state of emergency. Seasonality, short weekly cycles, and short-term market trends are factors that affect the price of fish. Species-specific impact estimates related to the COVID-19 pandemic can allow policymakers to implement recovery measures in a more targeted and effective manner. The results of our analysis can increase fishers’ and policymakers’ awareness of the usefulness of economic analyses and incentivize them to release data to establish a system to accumulate and analyze data strategically for urgent and appropriate interventions in the fisheries industry.

## Introduction

The rapid global spread of coronavirus disease 2019 (COVID-19) has led to unprecedented quarantine measures in most countries, and a major impact on both international markets and the Japanese economy, stifling the mobility of people and goods. Restrictions on economic activity to contain the pandemic aggravated the situation by decreasing demand and increasing uncertainty. Here, we focus on the impact on the fisheries industry. The accumulated effects of a rapid reduction in overseas demand and a slump in fish prices due to stagnant domestic demand are seen as negative demand shocks, as they resulted in a rapid contraction of demand in the fisheries and related industries (FAO 2020; Love et al. 2020; White et al. 2020). Moreover, fisheries that exploit living marine resources are exposed to a range of exogenous demand shocks in addition to catch uncertainty due to natural systems (Gephart et al. 2017).

Data collection, data accumulation, and scientific analysis are necessary to generalize demand shocks from an individual fisheries level to the industry as a whole (White et al. 2020). While the data can be used for informed policymaking, it can take time to accumulate such data and develop scientific approaches to make effective policies that address possible impacts (White et al. 2020). Furthermore, the data are mostly from commercial sources and not publicly available. There is currently no system in place in Japan to conduct such analyses systematically; consequently, policies to deal with demand shocks in the fisheries industry take more time to formulate.

Disaggregated data on landings and transactions are accumulated by cooperatives and local markets in the Japanese fisheries industry. To ensure effective policies to deal with demand shocks and to protect the Japanese fisheries industry in the face of the prolonged COVID-19 pandemic, it is necessary to collect and accumulate data promptly and utilize the information through rapid analysis for policy formulation. While accumulated data over a long period of time are required to ensure a scientific basis for an analysis, to date a dataset for such an analysis has not been compiled. To provide information for policies in a short time frame and with limited data on the market, this study proposes a method to analyze the impact from readily available data.

The study aims to present a quantitative understanding of domestic demand shocks in the Japanese fisheries industry due to the pandemic by using a counterfactual approach based on a time-series analysis of first-hand data from local markets to provide prompt information for policy formulation. While forecasting on what could happen is important for policymakers (Gecili et al. 2021), immediate measurement and feedback on the economic damage is also required to support vulnerable communities in the fisheries industry. To estimate the impact of the pandemic on the price of fish in local fisheries markets, we use the daily landing data provided by the Ishikawa Prefectural Federation of Fishermen’s Associations and forecast fish prices for 2020 assuming that COVID-19 had no impact on the industry. We then quantify the impact of COVID-19 by comparing the results with observed prices.

During the initial stages of the COVID pandemic in 2020, the impact of the outbreak on the Japanese fisheries industry was intuitively recognized by the industry and the authorities concerned (Research Institute for Humanity and Nature, “Sales of marine products due to new coronavirus infections down about 30 percent-With Corona, Supply Chain Reform is the Key to Reviving the Seafood Industry” [in Japanese]. https://www.chikyu.ac.jp/publicity/news/2020/0722.html. Accessed 23 Nov 2020). In Japan, the first COVID-19 case was reported on January 16, 2020, and the cumulative number of cases reached 100 and 1000 on February 21 and March 20, respectively. The government of Japan declared the first state of emergency on April 7 in seven major prefectures, including Tokyo and Osaka, and a nationwide state of emergency on April 16. The state of emergency did not require forced lockdowns, but large facilities were told to close their operations and restaurants to close earlier and cease serving alcohol. The initial decline in demand occurred due to a contraction in inbound tourist demand, and exports of high-grade fish decreased due to the restrictions on the international flow of people and goods (Ryukyu Shimpo “Lobster prices down 30% as seafood auction prices drop due to sharp drop in visitors to Japan, impact of new Corona” [in Japanese]. Ryukyu Shimpo) (Kameoka 2020). When Japan declared a state of emergency, the demand from the food service industry shrank significantly because of lockdowns. On the other hand, the demand for some species of fish increased because of an increase in demand for ingredients for home cooking (eating in) and the rise of direct sales on the Internet.

National and local governments have been providing comprehensive financial support for general economic activities in the form of subsidies to sustain businesses. While such comprehensive policy measures allow for a quick response, they also simplify the screening process for eligibility, which can lead to cases where recipients who do not need the benefits receive them. This trade-off between speed and accuracy can be improved by establishing a framework to understand the current situation by analyzing existing data instead of by collecting new data.

Using time-series analysis, we forecast the landing prices to estimate the effect and impact of policies and events. Wakamatsu (2014) quantified the economic effects of Marine Stewardship Council (MSC) certification in the Japanese fisheries industry by applying a structural break test and comparing fisheries with MSC certifications with those without and using landing data from Fukui Prefecture, Hyogo Prefecture, and the city of Maizuru. Sakai et al. (2018) estimated the impact of the Great East Japan Earthquake (henceforth, the earthquake) on oyster production by comparing affected and unaffected prefectures. Moreover, existing literature applies the regular time-series approach to find the cointegration of multiple variables to explore market or fishery structures (Helstad et al. 2005; Castillo-Manzano et al. 2014; Goto and Takanashi 2020). At the prolonged COVID-19 pandemic, the number of studies on the Japanese fisheries industry is limited. This study differs significantly from existing studies in that it utilizes time-series forecasting to assess the impact of COVID-19 on the unit price of fish.

The quantitative estimation of the impact of an event or a policy requires a case where the event did not happen, to make a comparison. For example, to measure the impact of COVID-19, we would want to compare a fishery impacted by COVID-19 with one with similar fishing patterns and environment unaffected by COVID-19 to identify the impact of the pandemic. If the objective variables are only compared before and after the event for a single fishery, it would not be possible to exclude other effects and quantify the intrinsic effects of the event itself. Similarly, unaccounted long-term trends of year-on-year comparisons may cause biased estimates. The year-on-year comparison cannot distinguish between seasonal cyclicality and year-specific effects because the control group is a single year. Thus, to evaluate with a degree of accuracy whether the year is abnormal relative to a normal year, it is essential to estimate the seasonality from a sufficiently long time series.

Ideally, we should compare fisheries affected and unaffected by COVID-19; however, it is almost impossible to find a fishery in Japan that has not been affected by COVID-19. Thus, in the counterfactual approach, we find a variable that is close to a fishery not affected by COVID-19 under certain assumptions and compare it with the corresponding variable of a fishery that was affected. This is referred to as the Rubin causal model (RCM) and is one of the basic frameworks used to estimate causal relationships (Rubin 2005). This approach makes it possible to estimate the impact of an event more accurately than simply comparing the target variables before and after an event. Thus, even for events with far-reaching societal impacts, such as the COVID-19 pandemic, it is possible to evaluate the difference using the counterfactual approach and to look for fisheries that really need assistance through an appropriate impact assessment, instead of simply looking at a before-and-after comparison.

Approaches that apply the RCM include randomized controlled experiments that experimentally compare groups that receive a certain treatment with those that do not, natural experimental approaches such as difference-in-differences, and regression discontinuity designs that compare groups unaffected by policy interventions or events after their occurrence with those that are affected. Given that the effects of the COVID-19 pandemic are universal, it is difficult to find a non-intervention group unaffected by the pandemic. Therefore, in this study, we simulate the unaffected state under certain assumptions. In other words, we assume that fish prices are determined by factors such as historical cyclical characteristics, trends, and recent prices and that the characteristics estimated from historical data would have persisted in 2020 had COVID-19 not occurred. Based on this assumption, price fluctuation patterns are estimated using pre-pandemic data, and these patterns are used as a virtual reality to “forecast” results for 2020. The difference between the latter and the actual value is then estimated as the impact of COVID-19. While this approach relies on stronger assumptions than those for other standard causal inference methods, it is definitely better than the year-on-year comparisons that are often used in the industry and in policy-making processes, and relatively simple to use, as it does not require costly experiments or suitable natural experiments.

## Materials and methods

### Data

In this study, we use data on daily landings in the ex-vessel market of Ishikawa Prefecture. The data record the daily quantity of fish landed in kilograms (kg), the landing value for each species, and fishing method at each fishing cooperative (branch office) in the prefecture. We use data from January 1 to June 7, 2020.

It is desirable to have data throughout the year to estimate the periodicity of the unit price. Hence, the weekly average price for the whole prefecture is calculated after aggregating the fishing methods and branch cooperatives by day and fish type. The average price is the summed value divided by the quantity. We use the aggregated data at a weekly level because daily data have consecutive days missing, which may cause autocorrelation if interpolated. Additionally, given a discrepancy between nominal and real prices due to the long-term time series, we obtain the real prices using the consumer price index (CPI) for 2015 supplied by the Statistics Bureau of Japan as the base price to adjust for inflation.

The Noto Peninsula, where Ishikawa Prefecture is located, is surrounded by the Sea of Japan and Toyama Bay. The Tsushima Current in the Sea of Japan and the Liman Current collide in Toyama Bay. Consequently, Ishikawa is endowed with a rich environment for fishing. In addition, the area has good access to the Yamato Bank, a shallow area in the middle of the Sea of Japan that is a productive fishing ground for major species. In this study, based on the number of landings, the characteristics of the fish, and the species unique to Ishikawa Prefecture, we selected five species: the Japanese flying squid (*Todarodes pacificus*), Japanese amberjack (*Seriola quinqueradiata*), red seabream (*Pagrus major*), blackthroat seaperch (*Doederleinia berycoides*, also called rosy seabass), and flathead flounder (*Hippoglossoides dubius* Schmidt). Squid fishing is one of the major fisheries in Ishikawa Prefecture. Small squid fishing boats annually land fresh squid from January to July, while medium-sized squid fishing boats fish offshore for frozen squid from June to December. The main method for bottom trawling in Ishikawa Prefecture is *yokake mawari* (single-layer trawl). Flathead flounder, red seabream, and blackthroat seaperch are all caught using the bottom trawling method. While most of the fisheries are in the area of Uchinoura, some also operate in the Sotoura area (from the Sea of Japan side). Purse seine fishing targets the Japanese amberjack, mackerel, and horse mackerel. Fixed nets catch a wide variety of fish, including squid and red seabream, but it is particularly effective for catching Japanese amberjack.

## Method

In this study, we construct a time-series model that incorporates price fluctuations and time-series trends immediately before the pandemic, in addition to the seasonal cycle before March 2020 (when the impact of COVID-19 became apparent in Japan), to understand the quantitative impact of the demand shocks caused by the pandemic. By calculating the difference between realized fish prices during the COVID-19 pandemic and counterfactual prices, we quantitatively estimate the demand shocks in the fisheries industry during the pandemic.

Because all the information used for the counterfactual simulations is before the COVID-19 outbreak in Japan, the predicted fish prices are based on past seasonality and cyclicality, and recent price fluctuations. To use this forecast as a counterfactual, it is necessary to make a few assumptions. First, the possible confounding factors do not undergo a large change in their pattern across the pre-COVID period so that seasonality and cyclicality capture the patterns. Second, there are no year-specific events after March 2020 other than the COVID-19 outbreak. In other words, we conclude that the difference between the simulated and actual prices is due to the impact of the COVID-19 outbreak. We are unable to identify other possible factors specific to 2020. This differs from other approaches that use control groups in a framework to analyze a policy or a shock as a natural experiment when an event occurs for only a subset of groups. These methods use groups that are not affected by the policy or event as counterfactuals (e.g., prefectures that are adjacent to the prefecture that enacted an ordinance) to identify factors other than intervention that could change the variables and the changes caused by such intervention. As mentioned earlier, since COVID-19 has a far-reaching impact on society, it is difficult to find such non-affected groups. It should be noted that, because of the limitations of the data and the event, strong assumptions are made.

Problems arise while conducting the time-series analysis when there are many missing values in the data. In the case of the fisheries industry, which is associated with a great deal of seasonality, there are periods with no landings at all, depending on the fish species. The fish species covered in this study are landed on a yearly basis, and there are no long periods without landings, although there are some days without landings, such as New Year’s Day and during the summer holidays, which last for a few consecutive days. Hence, we use linear interpolation to interpolate the prices.

We adopt a dynamic harmonic regression (DHR) model with seasonality for forecasting (Hyndman and Athanasopoulos 2018) as the first choice of the model and the seasonal autoregressive integrated moving average (SARIMA) model if the DHR model is unable to capture the autocorrelation of the price time series. The DHR model uses the Fourier series to represent multiple periodicities, such as a weekly cycle due to the days of the week and annual seasonality. The effect of price autocorrelation is then captured by fitting an autoregressive integrated moving average (ARIMA) structure to the model errors. The SARIMA model, often used in time-series models that take seasonality into account, is computationally costly when considering long-term periodicity in high-frequency data because it uses many lag terms to represent seasonality. Because the DHR model expresses long-term periodicity by using a Fourier series and estimating the Fourier coefficients, it is possible to estimate a periodic time-series model with a minimum number of parameters. We apply the SARIMA model to the data of some species when the portmanteau test indicates that the residuals of the DHR are still autocorrelated. These cases indicate that the seasonality is not well explained by the harmonic form that is expressed in a Fourier series.

The general autoregressive moving average (ARMA) model can be formulated as follows

$$e_{t} = c + \mathop \sum \limits_{i = 1}^{I} \varphi_{i} e_{t - i } + \varepsilon_{t} + \mathop \sum \limits_{j = 1}^{J} \theta_{j} \varepsilon_{t - 1},\ \varepsilon_{t} \sim N\left( {0,\sigma^{2} } \right),$$

where ${\mathrm{e}}_{\mathrm{t}}$ is the time-series data in period $t$, and $c$ is a constant. ${\upvarepsilon }_{\mathrm{t}}$ is the normal white noise at period $t$ and the magnitude of its variance is ${\upsigma }^{2}$, while $\upphi$ and $\theta$ are the coefficients of the autoregressive term and the moving average term, respectively, $\mathrm{I}$ and $J$ are the orders of the autoregressive term and the moving average term, respectively. An ARMA model that uses $D$-order difference series as the target time-series data is called an ARIMA model. We employ the ARIMA model with the first-order difference time series.

The DHR model adopted in this study is formulated as follows

$$p_{t} = a + \mathop \sum \limits_{k = 1}^{K} \left\{ {\alpha \sin \left( {\frac{2\pi kt}{{52}}} \right) + \beta \cos \left( {\frac{2\pi kt}{{52}}} \right)} \right\} + e_{t} ,$$

where ${p}_{t}$ is the unit price in period $t$, and $a$ is the constant term. In this study, annual periodicity is incorporated into the model, and $\mathrm{\alpha }$ and $\beta$ are the coefficients of their respective Fourier series terms. The seasonality is captured by the Fourier series, which is a series of Fourier terms, sine and cosine. Because the periodic cycle is a year and the data are at a weekly level, we choose $2\pi /52$ as a coefficient in the Fourier term. $\mathrm{K}$ is the parameter that specifies the smoothness of the function approximation by the Fourier series. The parameter $\mathrm{K}$ must be an integer smaller than the number of points in the cycle divided by 2, so it must be less than 26. In general, the smaller the $\mathrm{K}$, the smoother the periodicity, and the larger the $\mathrm{K}$, the more complex the periodicity. $\mathrm{I},\mathrm{ J}$, and $K$ were chosen to minimize the Akaike information criterion (AICc); $I$ and $J$ vary from 1 to 10, and $K$ varies from 1 to 20. The model, estimated by maximum likelihood estimation, uses the forecast package in R (Hyndman and Athanasopoulos 2018; Hyndman et al. 2020). Hyndman and Khandakar’s method was used to determine the orders of the ARIMA model, and the brute force method was used to determine $\mathrm{K}$ so that the AICc is minimized (Hyndman and Khandakar 2008).

To assess the forecasting capability of the model, we evaluate the forecasting accuracy on the test data. As the test data, we separate every quarter for the period 2018–2019. The model is estimated using the training data from the beginning of the period to immediately before the test data. A quarter of a year is close to the period affected by COVID-19 in the actual data, from March 1 to June 7, 2020.

We then calculate the prediction indices, mean error (ME), and mean percentage error (MPE). Generally, root mean square error (RMSE) and the mean absolute error (MAE) are used to evaluate the forecasting model because they give the absolute difference between the predicted and measured values. However, these values are large in our data because weekly fluctuations cannot be predicted perfectly. Therefore, since the objective of this study is to ascertain a short-term trend due to the impact of the COVID-19 pandemic, we consider ME and MPE, which have smaller values when the variations of the measured values against the predicted values cancel each other out, as suitable for evaluation.

## Results

**Fig. 1:**
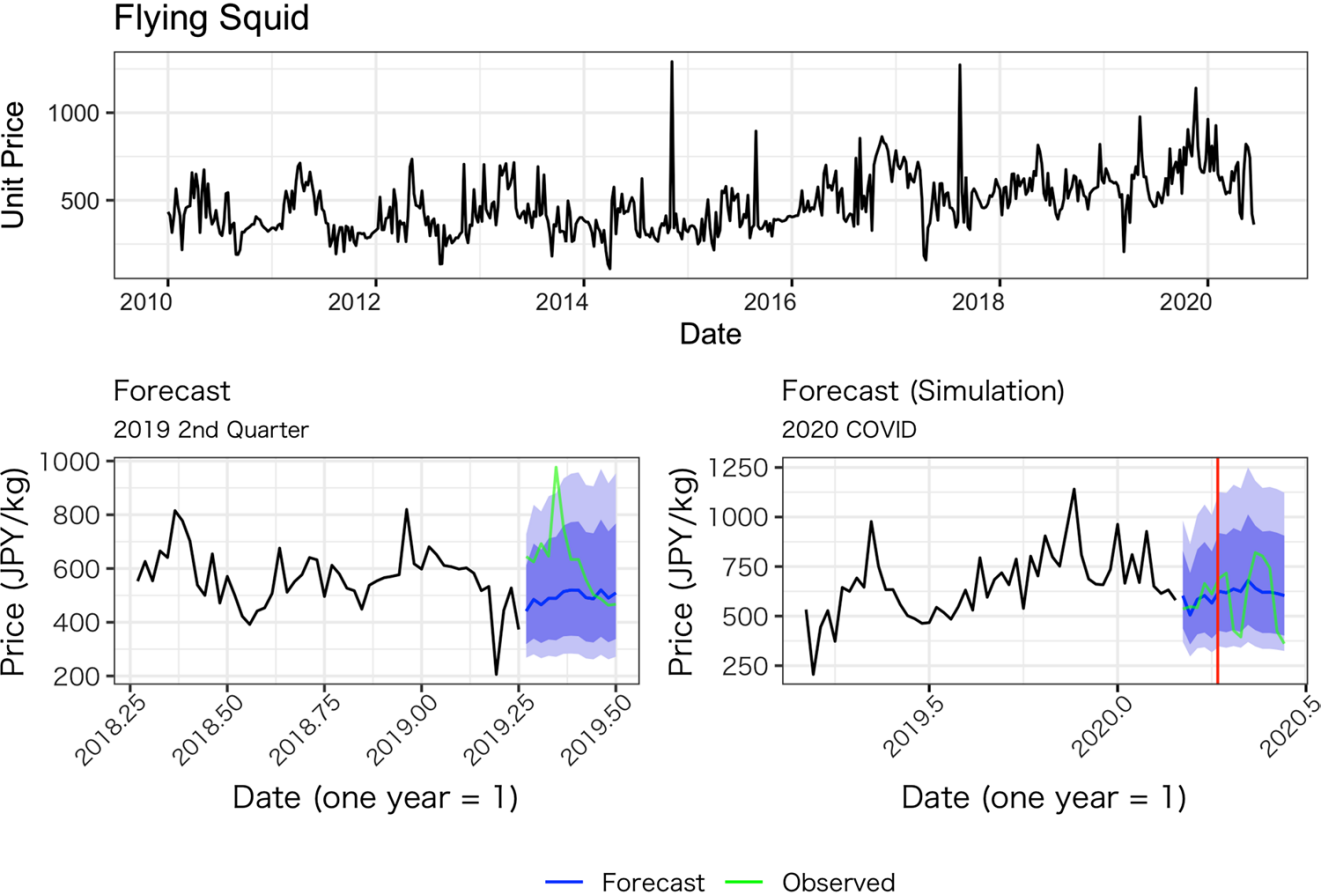
Observed daily average prices for Japanese flying squid (top panel) and the comparison between the model’s prediction and observed weekly average prices (bottom left for 2019, bottom right for 2020)

**Fig. 2:**
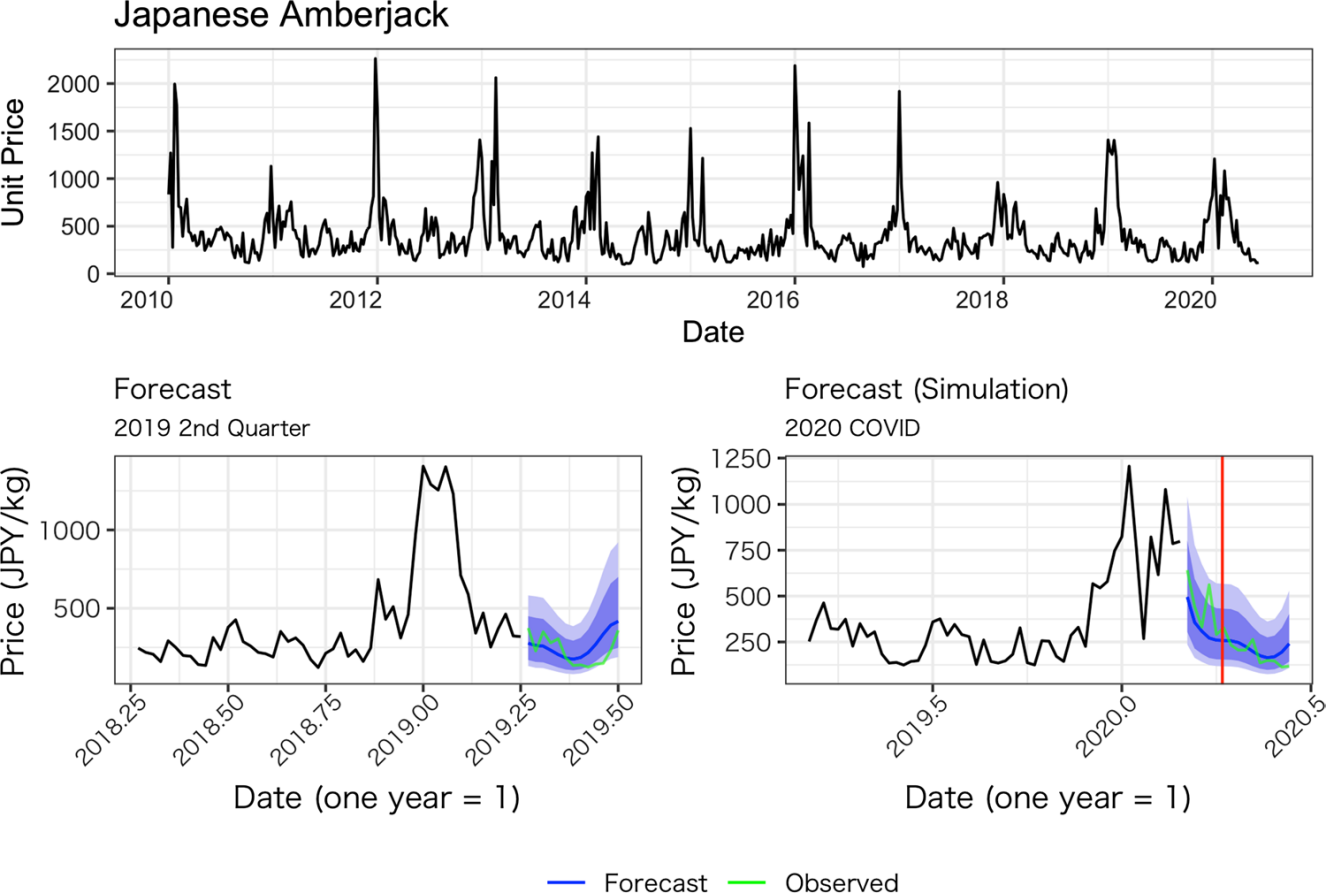
Observed weekly average prices for Japanese amberjack (top panel) and the comparison between the model’s prediction and observed weekly average prices (bottom left for 2019s quarter, bottom right for 2020 after COVID-19 pandemic)

**Fig. 3:**
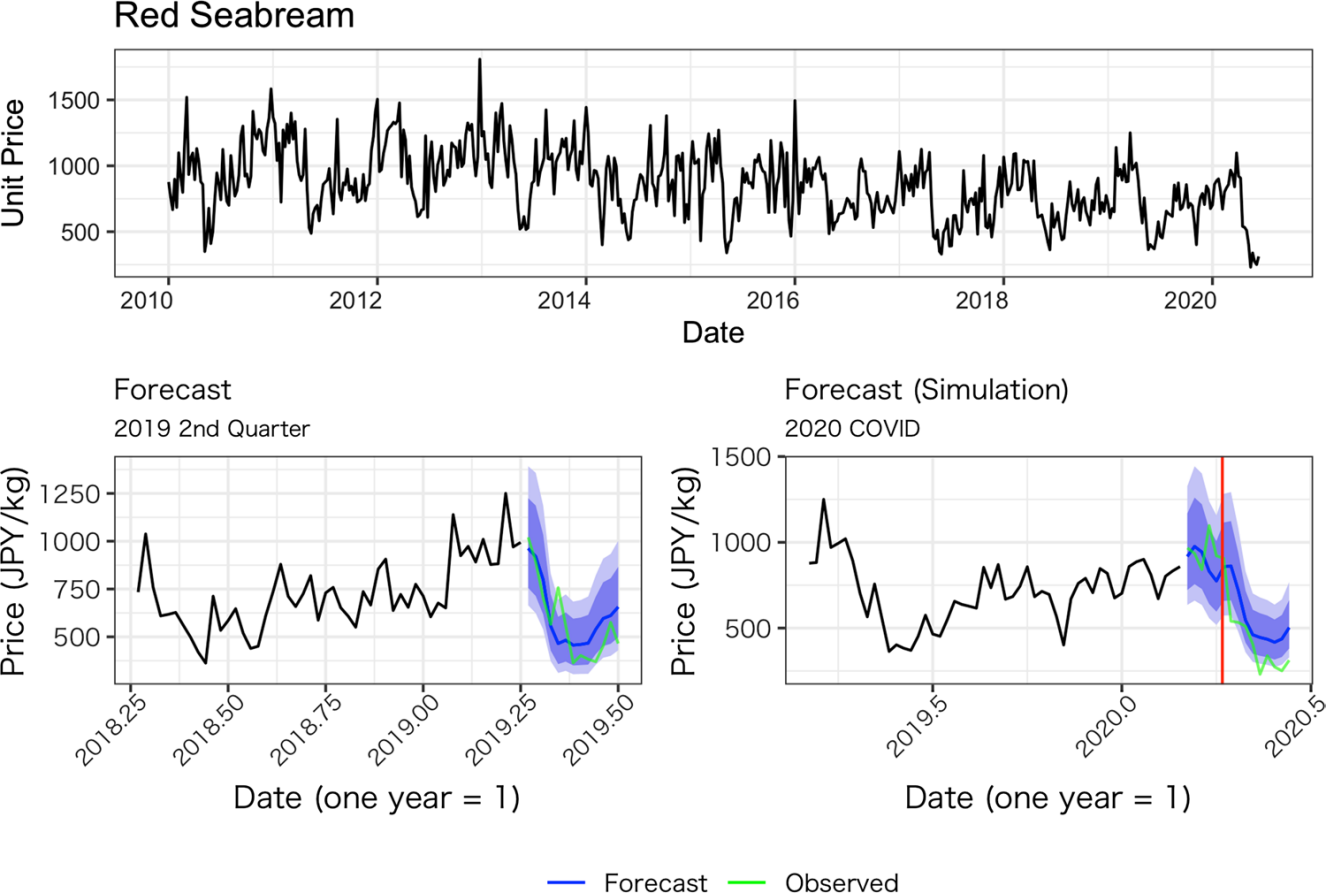
Observed weekly average prices for red seabream (top panel) and the comparison between the model’s prediction and observed weekly average prices (bottom left for 2019, bottom right for 2020)

**Fig. 4:**
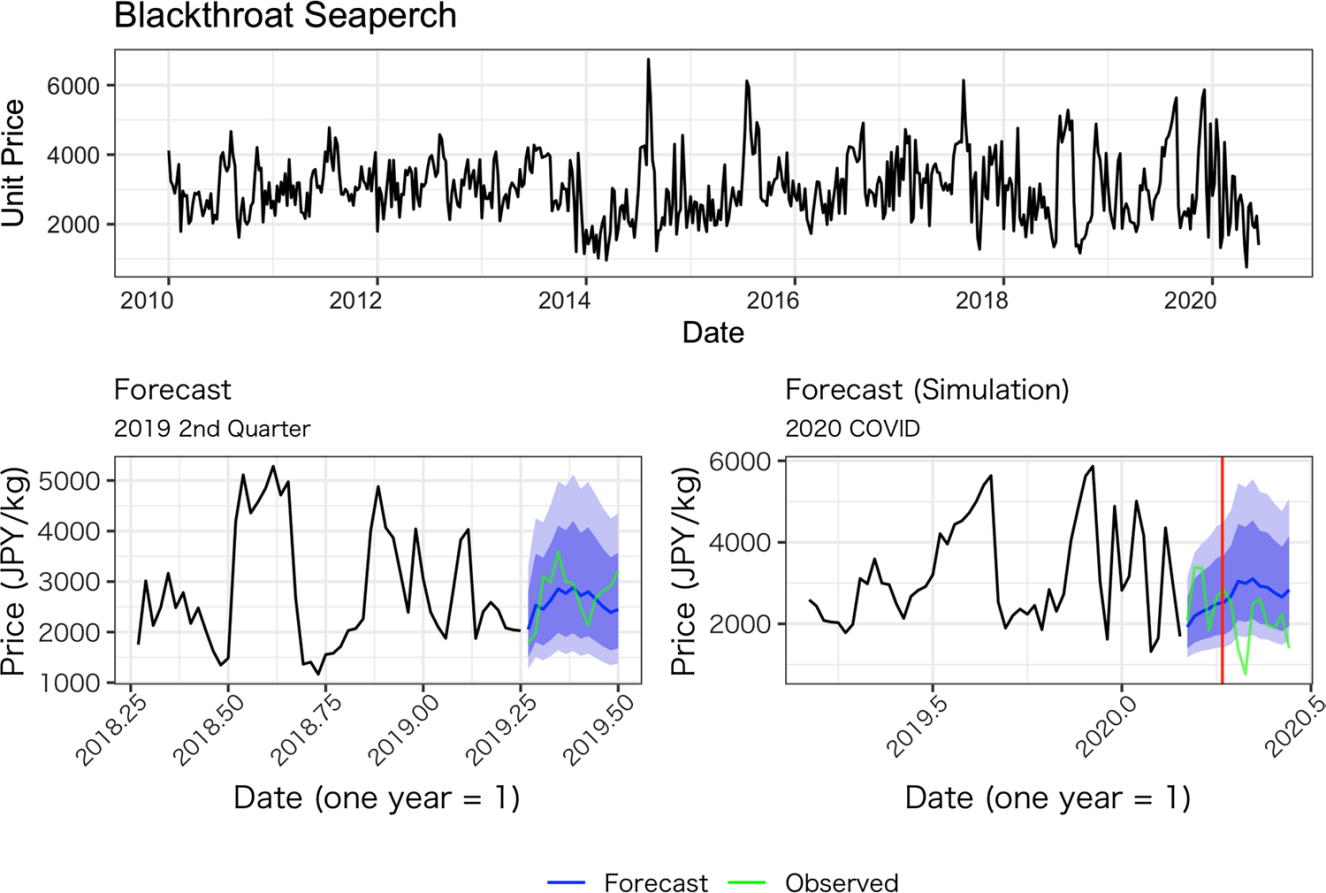
Observed weekly average prices for blackthroat seaperch (top panel) and the comparison between the model’s prediction and observed weekly average prices (bottom left for 2019, bottom right for 2020)

**Fig. 5:**
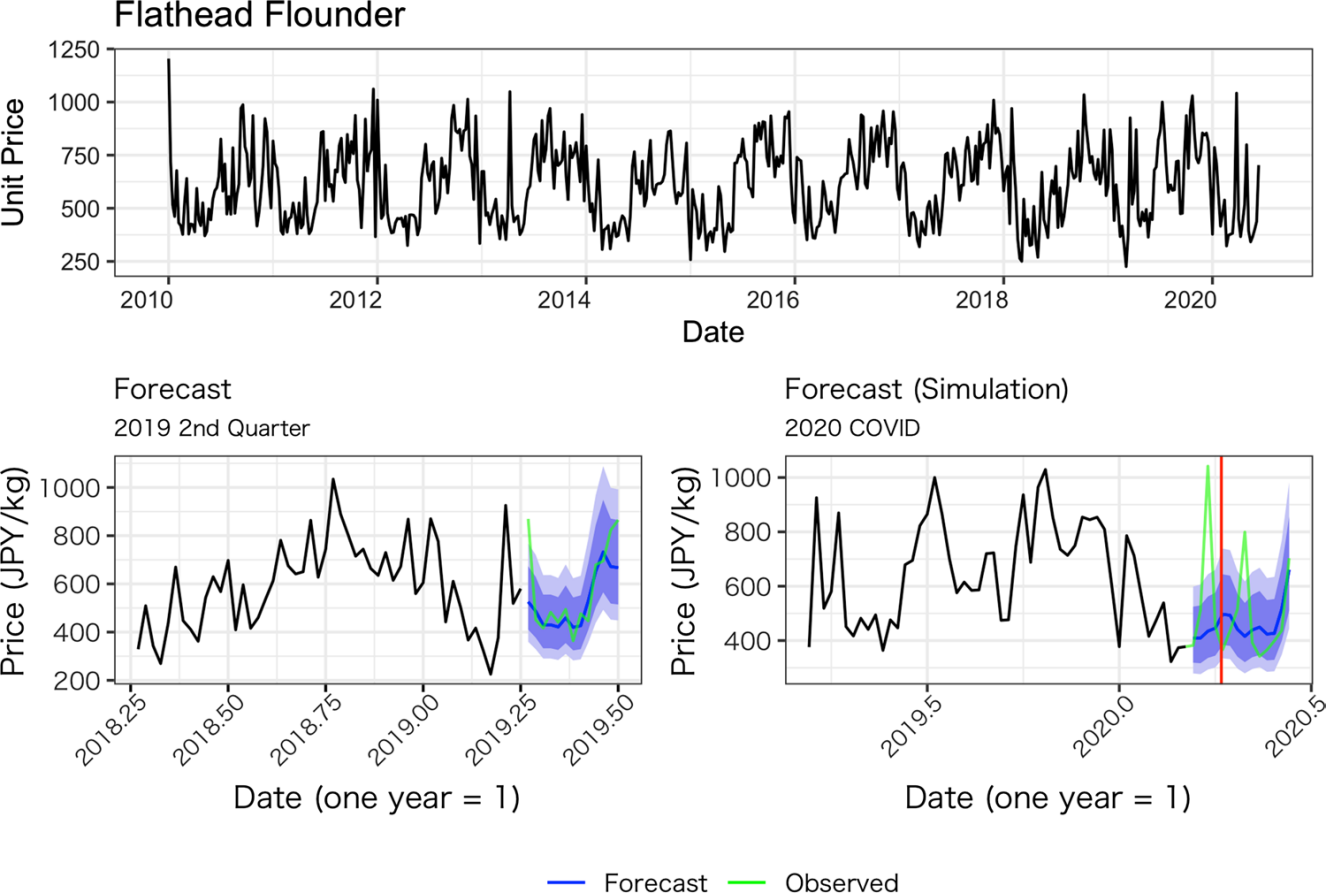
Observed weekly average prices for flathead flounder (top panel) and the comparison between the model’s prediction and observed weekly average prices (bottom left for 2019, bottom right for 2020)

Figures 1, 2, 3, 4 and 5 show the average weekly unit price data for each fish species and a comparison of the results of the model’s predictions with the actual measurements. In each figure, the upper panel shows average weekly prices for the entire data period (2010 week 1 to 2020 week 24). On the one hand, the unit prices of all fish species display seasonal fluctuations; on the other hand, the fluctuations are not always the same every year: some years show a fast decrease after an increase, while other years do not. Based on these observations, we believe the model used in this study is appropriate because it incorporates both seasonality and recent price trends.

The results of the model estimations and the statistical tests for each fish species are shown in Table 1. First, we see that the results of the augmented Dickey–Fuller (ADF) test reject the null hypothesis that there is a unit root in the time-series data of the weekly unit price of the first-order difference for all years and fish species. With this result, we adopt the alternative hypothesis of stationarity and assume that it is reasonable to use a DHR model that assumes stationarity. The DHR model is applied for Japanese amberjack and red seabream. The smoothing parameter, K, is 6 for Japanese amberjack and 13 for red seabream, suggesting that seasonality is relatively simple for Japanese amberjack. For the other three species, the Ljung–Box (LB) test rejected the null hypothesis that the residuals are not autocorrelated under the DHR model; hence, we apply the SARIMA model (Table 1, columns 13–15). The LB test results show that the null hypotheses are rejected at the 5% level only for flathead flounder. We use the model for the prediction as it is close to the threshold, but we admit that this is a potential concern.

**Table 1:** Result of model estimations and tests

Species	Model	p	d	q	P	D	Q	AICc	K	ADF stat	ADF *p* value	LB df	LB stat	LB *p* value
Japanese flying squid	SARIMA	1	1	4	0	0	1	82.34		–4.41	0.01	77	83.87	0.28
Japanese amberjack	DHR	5	1	1				415.09	6	–5.49	0.01	65	67.16	0.40
Red seabream	DHR	0	1	9				–151.33	13	–5.12	0.01	48	57.39	0.17
Flathead flounder	SARIMA	2	0	3	0	1	2	–131.30		–4.32	0.01	76	99.40	0.04
Blackthroat seaperch	SARIMA	2	0	5	1	0	0	–24.29		–6.65	0.01	74	64.59	0.77

ADF = augmented Dickey–Fuller test; LB = Ljung–Box test; AICc = Akaike information criterion with a correction for small sample sizes

**Fig. 6:**
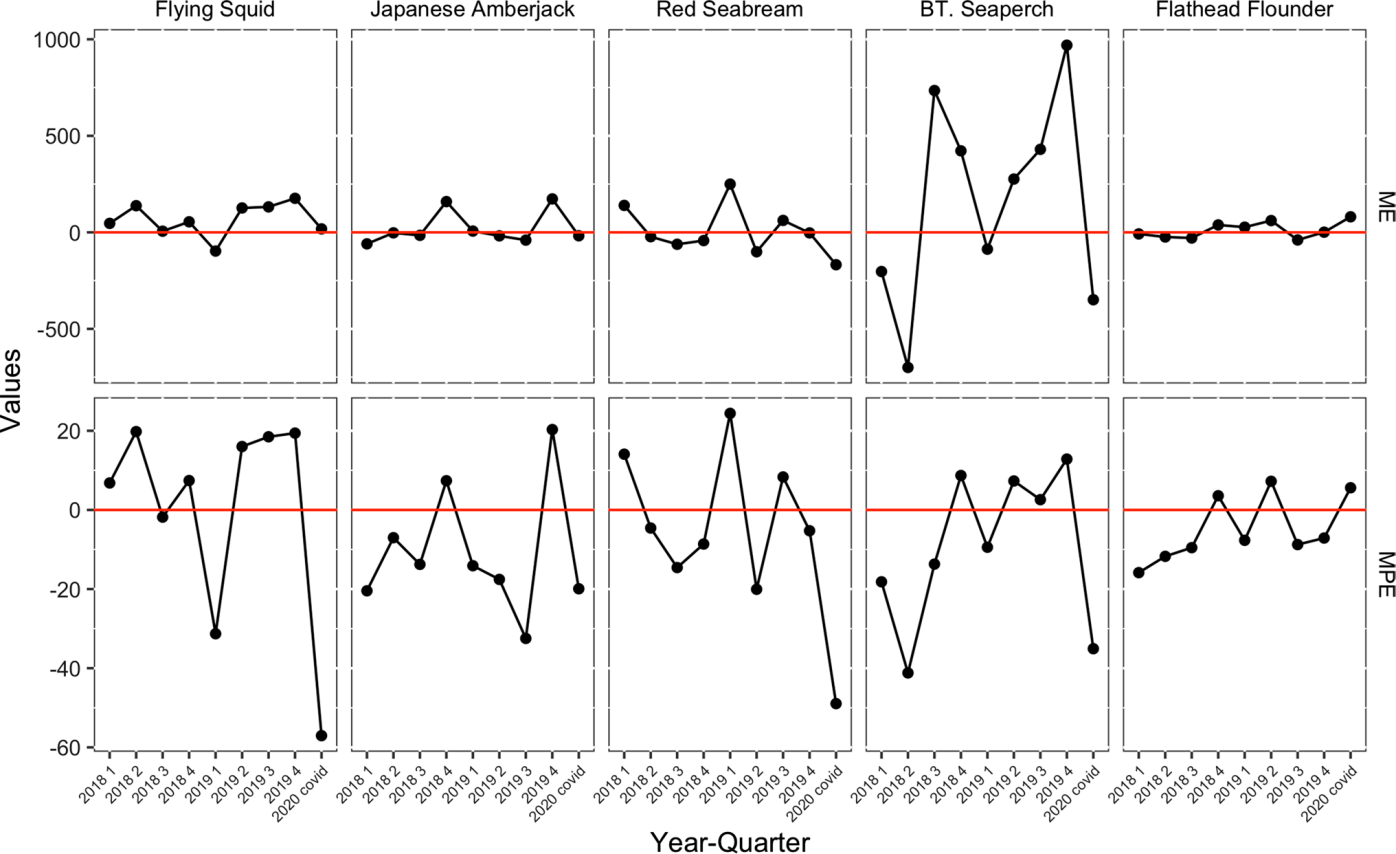
Mean errors and mean percentage errors of the predictions evaluated on the test data sets

The performance of our predictions can be seen in the results of the predictions for the second quarter of 2019, as presented in the lower-left panel of Figs. 1, 2, 3, 4 and 5. These figures show that the predictions reproduce the approximate trend of price fluctuations. For the five fish species selected, the weekly variation in the observed average unit price moves around the predicted trend (blue line), and although it is not possible to fully predict the weekly variation, it predicts the short-term trend. However, in the case of Japanese flying squid, there is a period in the second quarter of 2019 when the trend itself shifts away from the predicted value, which may deviate from the predicted trend as it moves away from the starting point of the forecast. Figure 6 shows the mean errors and mean percentage errors of the predictions for each test set and the COVID-19 period. Mean errors are small overall except for blackthroat seaperch. Blackthroat seaperch is a high-grade fish; average prices are high because of its high value. In addition, the demand for the species can fluctuate because it is a relatively new species in the domestic market. The mean percentage error results show that the errors are mostly within the 20% range, but the errors during the COVID-19 period deviate from those for species such as the flying squid, red seabream, and blackthroat seaperch, indicating the peculiar impact of COVID-19.

**Fig. 7:**
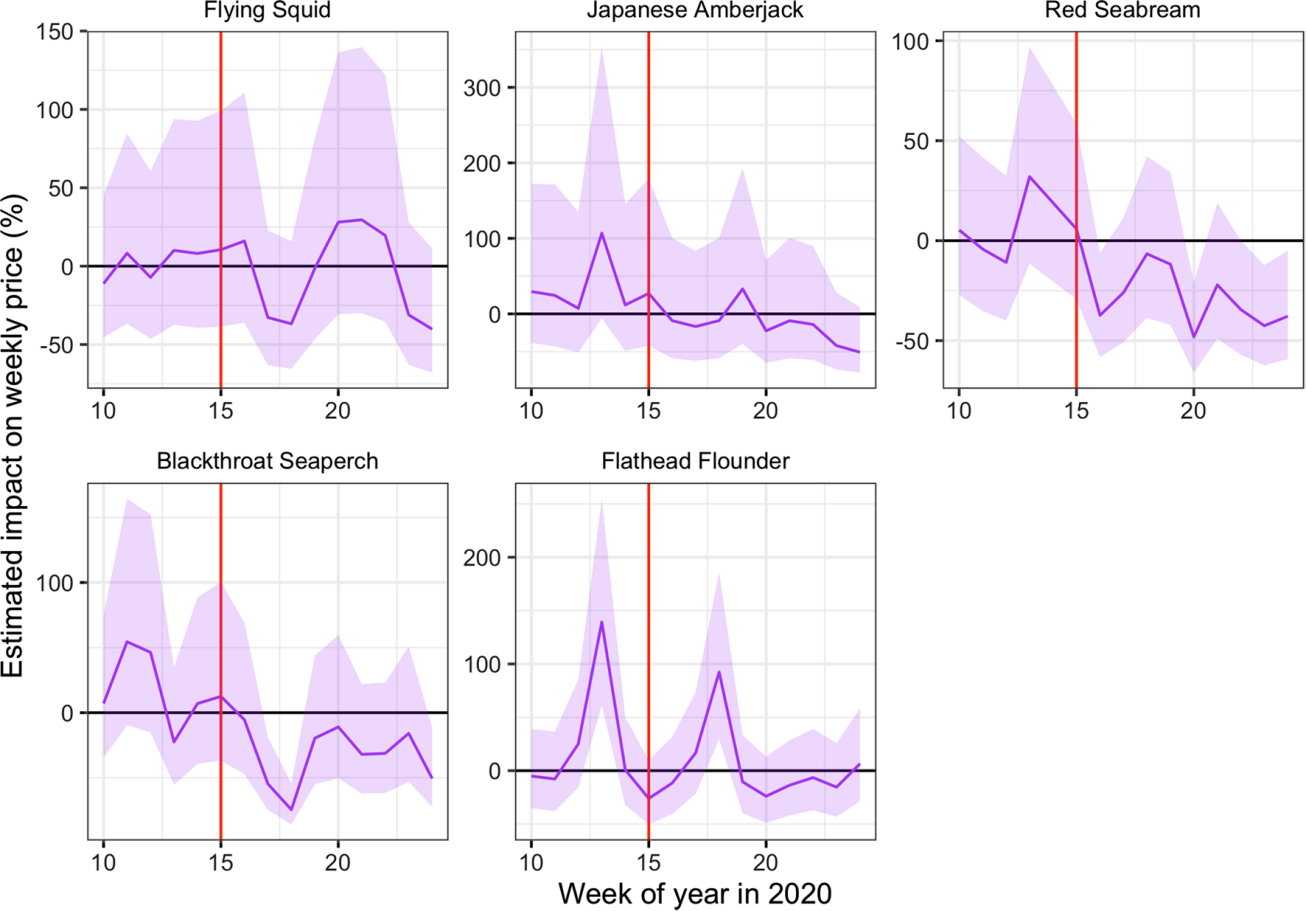
Estimated impacts on the weekly prices from week 10 (the week of March 2, 2020) to week 24 (the week of June 1, 2020). The red vertical line indicates the week when the first state of emergency was declared (week 15, April 7)

Figure 7 shows the estimated impact of the COVID-19 pandemic on the weekly prices for each species. The species can be divided into two categories based on the trends of the impact, namely fish species wherein prices did not change significantly and fish species wherein prices were significantly lower. The pandemic had no significant impact on the price of Japanese flying squid, Japanese amberjack, and flathead flounder: –12.8 JPY (–2.01%), 23.2 JPY (4.51%), and 43.6 JPY (10.7%), respectively (Table 2). The price of the Japanese amberjack appeared to drop significantly after the government’s declaration of a state of emergency, as indicated by the red line in the figures; however, this was largely due to seasonal fluctuations and was not a significant deviation from the predicted range. The weekly price of flathead flounder had two spikes—before and after the state of emergency declaration—but a large price drop was not observed.

**Table 2:** Average estimated impacts of COVID-19 pandemic on the ex-vessel prices

Species	All terms		After week 15	
	Impact	Impact (%)	Impact	Impact (%)
Flying squid	–12.76	–2.01	–23.10	–3.83
Japanese Amberjack	23.16	4.51	–23.03	–11.25
Red seabream	–70.74	–14.64	–137.94	–26.08
Blackthroat Seaperch	–416.90	–12.65	–829.14	–28.22
Flathead flounder	43.62	10.67	–0.55	0.78

Large negative impacts are estimated for the price of red seabream and blackthroat seaperch. In the case of red seabream, for which average prices also tend to be subject to seasonal fluctuations, the observed prices in 2020 are noticeably lower than the forecasted prices after the declaration of the state of emergency (Fig. 3). The estimated average price for red seabream price is –70.7 JPY (–14.6%) and even lower after the state of emergency. Blackthroat seaperch prices dropped significantly relative to the simulated price after the state of emergency. The average estimated impact is –416.9 JPY (–12.65%), and it does not recover as high as the simulated price (Fig. 7).

**Fig. 8:**
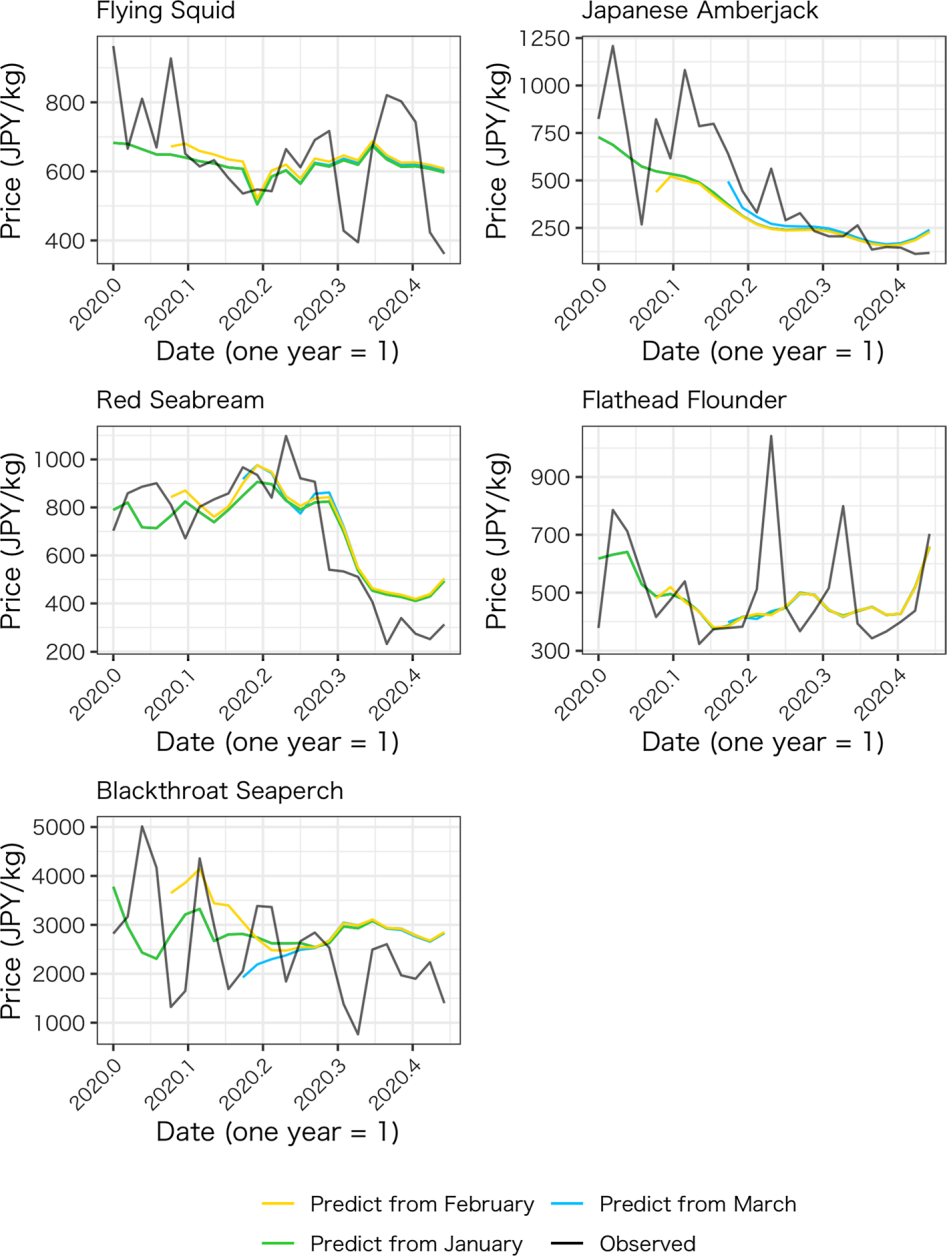
Comparison of the simulations by different cutoff dates of periods under COVID-19 pandemic

A possible concern with our approach is the arbitrary cutoff date of the post-COVID term, which is March 1 in our main estimation. It is apparent that the COVID-19 pandemic started to impact society around the beginning of March. For example, the Hokkaido Prefecture, Japan’s northernmost prefecture, declared a state of emergency on February 28, 2020, ahead of the national decision (“Hokkaido declares state of emergency over coronavirus” [in Japanese]. Kyodo News). To check the robustness of the result, we simulate the counterfactual prices using the different cutoff dates. In addition to the main simulation (March 1 as the start date for the impact of COVID-19), February 1 and January 1, 2020 are also used as start dates for the period under the impact of the COVID-19 pandemic. Figure 8 shows that the simulated prices are not sensitive to the selection of start dates for most of the species except for blackthroat seaperch. The simulated prices for blackthroat seaperch differ depending on the start date, but it converges toward the end of the period. The SARIMA model captures the effect of the prices in the preceding weeks with the lag terms, and how the highly volatile prices for blackthroat seaperch in the preceding weeks affect the simulated prices immediately after the prediction term begins. However, these volatile prices do not affect the mid-term simulation as the simulated prices converge.

## Discussion

**Fig. 9:**
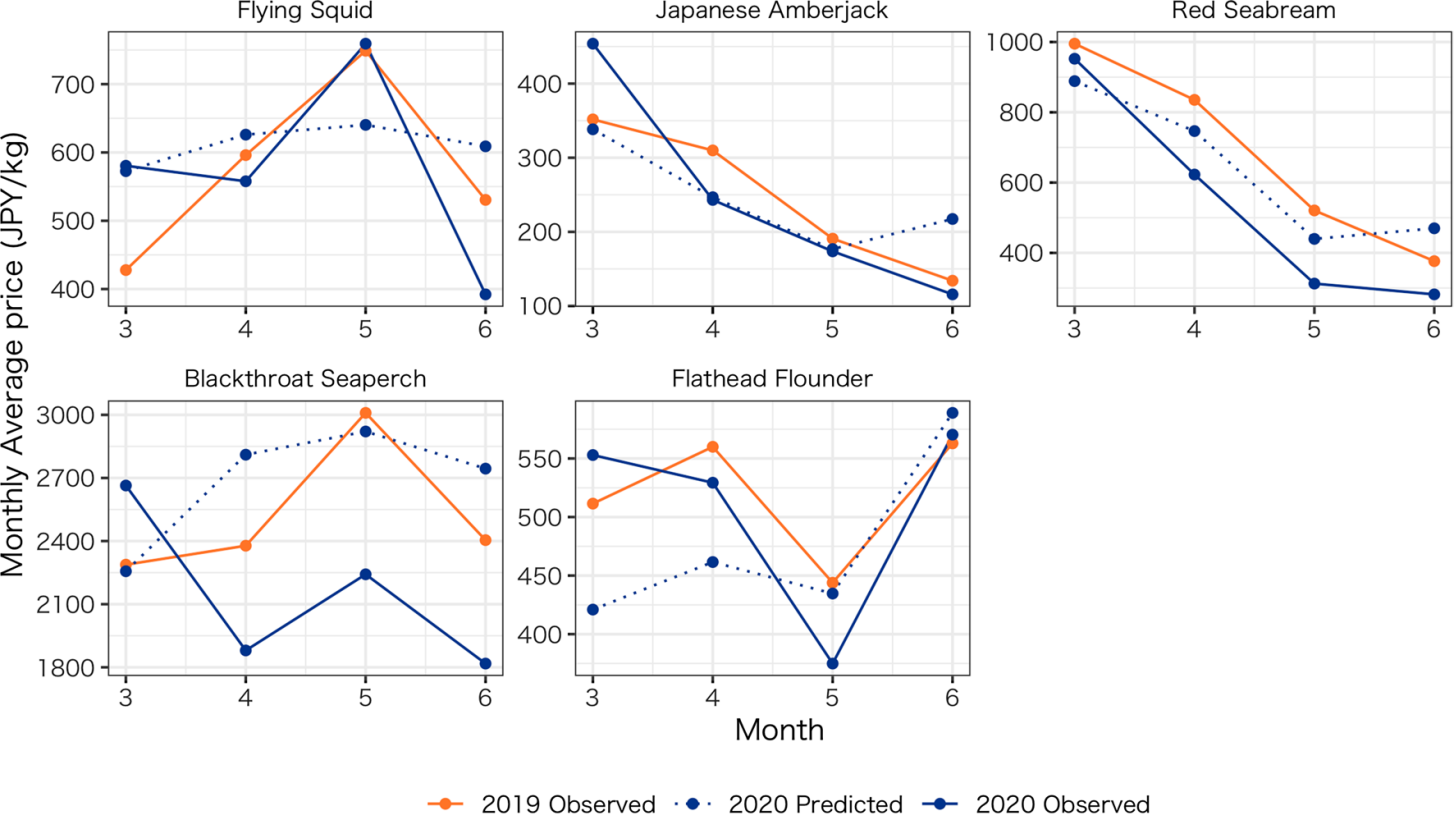
Monthly averages of the observed and forecasted prices

Note that a simple before-and-after comparison of the price trends in 2020 shows that prices for some fish species had decreased, but this decline can be attributed to seasonality (e.g., Japanese amberjack). This cyclicality is the basis for using year-on-year comparisons in practice. Figure 9 shows observed monthly average unit prices for 2019 (orange line) and 2020 (navy line) and the monthly average of the predicted prices for 2020 (dotted line) applied for a year-on-year comparison of the same month. In this study, the data for 2020 are limited to only 7 days in June for comparison. In the case of Japanese flying squid and Japanese amberjack, the observed average prices for 2019 and the predicted prices for 2020 for the same month are close overall, but the observed price of squid in March 2019, for example, is lower than the predicted price for 2020. Therefore, a year-on-year comparison of prices for the same month may lead to an assessment that the average price of squid had increased, but the counterfactual approach reveals that the price would have increased without the COVID pandemic and subsequent measures, and hence the price had not increased.

For red seabream, the observed monthly average unit price for the 2019 trend (orange line) is similar to the forecasted value for 2020 (dotted line). However, the magnitude of the impact may be overestimated if the year-on-year comparison is used because the observed price in 2019 is greater than the forecasted price for 2020, which takes into account the recent trend of the data. In the case of blackthroat seaperch, there is a large disparity between the monthly price for 2019 and the predicted price for 2020. In April 2020, the predicted price was much higher than the observed price for 2019. In this case, a year-on-year comparison could underestimate the impact.

For flathead flounder, the average monthly price in 2019 is higher than the forecast value, and the year-on-year comparison may overestimate the extent of the decline. As described above, the year-on-year change, which includes effects specific to 2019 but not recent trends in 2020, may deviate from a forecast that incorporates past cyclicality and recent trends. Thus, although a year-on-year comparison is a simple assessment, it is inferior to the method proposed in this study in terms of the capability of the impact evaluation.

The study reveals that the speed of fish markets’ response to COVID-19 varied by fish species. The results of this study have key implications for fisheries policy, such as in the case of the impact of COVID-19. The considerable variability among affordable and high-quality fish requires different policy strategies. High-end fish such as red seabream and blackthroat seaperch are in high demand in luxury restaurants that have been greatly affected by the declaration of a state of emergency, while fish such as Japanese amberjack, a general consumer preference, was seemingly less affected. From April to June, when the unit prices for Japanese amberjack and red seabream were low, there was a difference in how much the prices fell for each species. For red seabream, the rate of decline was even lower than the predicted downward trend due to seasonality and other factors.

While seasonality is a major factor affecting seafood prices, short-term market trends also play a crucial role. The DHR and SARIMA models incorporate these factors and are seen to be very effective in predicting price trends in the absence of irregular events. The advantage of these models in predicting the counterfactual (i.e., a simulation of the future that would have happened without an event such as the COVID-19 pandemic) lies in the realistic prediction using recent information. This is a large advantage over year-on-year comparisons, which does not take into account any new information from the later year.

Methods for calculating counterfactuals as simulations of what might have happened have been developed and applied in recent years. They produce more plausible counterfactuals while accurately estimating effects or impacts with the right circumstances and design. While the proposed method is simple and more plausible than the ad hoc year-on-year comparison used in practice, we note the potential issues and the direction of future research. Our model estimates the aggregated impact of the COVID-19 pandemic but does not identify the specific channel. For example, the direct impact on price due to the decline in demand for species A and the indirect impact through the price of species B are not separated if the prices of species A and B are correlated. In addition, the model does not consider the heteroskedasticity of the price data, which may affect the estimates of the prediction interval. Ideally, impact estimates that consider demand structure should be used to simulate the counterfactual using the demand equations. For instance, (Gordon 2021) simulates price and revenue outcomes under an alternative policy due to the COVID-19 pandemic for Canadian lobster and snow crab fisheries using a model constructed in a pre-COVID study (Gordon 2020).

To construct more detailed empirical economic models to evaluate the impact on market prices, detailed long-term data accumulation is necessary. Japan has a long history of first-hand local markets where fishermen’s landings are traded quickly and mainly by fishery cooperatives. While data have been accumulated, they have not yet been utilized. A possible way to promote the use of these data is to demonstrate the usefulness of the existing data through the best possible analyses and to spread the idea to those who own the data.

The accumulation of data requires strategic promotion in the future. In this study, with the cooperation of Ishikawa Prefecture, we use weekly data to observe price fluctuations that could not be captured by monthly averages. To determine whether the short-term fluctuations in prices within a month are cyclical or due to shocks, the trend predicted using the time-series model can be compared with the actual observed values. While such data are frequently recorded and accumulated in the fisheries industry, there is no system or rule for using them for research and analysis. In addition to being instrumental in assessing the impacts of COVID-19, we believe that rapid and valid analyses will become even more important in the future in the fisheries industry, which is constantly exposed to uncertainty. Further, the fisheries industry needs to establish an effective strategic data accumulation and utilization system. While some countries have centralized institutions to collect fisheries market data (e.g., sales organization in Norway; Pettersen 2016), Japan’s fisheries cooperatives are largely decentralized. To promote strategic data collection under such circumstances, fishers and those related to the industry should be incentivized to collect and provide data. Thanks to the recent development of information and communication technology (ICT), data collection and utilization are more accessible (Wada et al. 2010) (Pescadata, https://pescadata.org/. Accessed 15 Jul 2021). However, to incentivize fishers to provide data, they need to be aware of the usefulness of analyses using the data collected. As more and detailed data become available, more detailed analyses can be made to inform fishers, which should incentivize fishers further. Our analysis is the first step to drive this cycle to improve fisheries management and the industry.
