# Correction: Evaluating the impact of COVID-19 on ex-vessel prices using time-series analysis

**DOI:** 10.1007/s12562-023-01688-4

**Published:** 2023-03-21

**Authors:** Keita Abe, Gakushi Ishimura, Shinya Baba, Shota Yasui, Kosuke Nakamura

**Affiliations:** 1 Centre for Applied Research at NHH, Helleveien 30, 5045 Bergen, Norway; 2 Iwate University and National Institute of Environmental Studies, Morioka, Japan; 3 Logics of Blue, Hyogo, Japan; 4 CyberAgent, Inc, Tokyo, Japan; 5 Iwate University, Morioka, Japan

**Correction to: Fisheries Science (2021) 88:191–202** 10.1007/s12562-021-01574-x

The article “Evaluating the impact of COVID-19 on ex-vessel prices using time-series analysis”, written by Keita Abe, Gakushi Ishimura, Shinya Baba, Shota Yasui, Kosuke Nakamura, was originally published Online First without Open Access. After publication in volume 88, issue 1, page 191–202 the author decided to opt for Open Choice and to make the article an Open Access publication. Therefore, the copyright of the article has been changed to © The Author(s) 2023 and the article is forthwith distributed under the terms of the Creative Commons Attribution 4.0 International License, which permits use, sharing, adaptation, distribution and reproduction in any medium or format, as long as you give appropriate credit to the original author(s) and the source, provide a link to the Creative Commons licence, and indicate if changes were made. The images or other third party material in this article are included in the article's Creative Commons licence, unless indicated otherwise in a credit line to the material. If material is not included in the article's Creative Commons licence and your intended use is not permitted by statutory regulation or exceeds the permitted use, you will need to obtain permission directly from the copyright holder. To view a copy of this licence, visit http://creativecommons.org/licenses/by/4.0/

